# Augmenting Sulfur Metabolism and Herbivore Defense in *Arabidopsis* by Bacterial Volatile Signaling

**DOI:** 10.3389/fpls.2016.00458

**Published:** 2016-04-08

**Authors:** Mina Aziz, Ranjith K. Nadipalli, Xitao Xie, Yan Sun, Kazimierz Surowiec, Jin-Lin Zhang, Paul W. Paré

**Affiliations:** ^1^Department of Chemistry and Biochemistry, Texas Tech University, LubbockTX, USA; ^2^Center for Plant Lipid Research, University of North Texas, DentonTX, USA; ^3^College of Pastoral Agriculture Science and Technology, Lanzhou UniversityLanzhou, China

**Keywords:** plant growth-promoting rhizobacteria (PGPR), *Bacillus amyloliquefaciens* GB03, bacterial volatile organic compounds (VOCs), glucosinolates (GSL), sulfur assimilation, plant-defense priming

## Abstract

Sulfur is an element necessary for the life cycle of higher plants. Its assimilation and reduction into essential biomolecules are pivotal factors determining a plant’s growth and vigor as well as resistance to environmental stress. While certain soil microbes can enhance ion solubility via chelating agents or oxidation, microbial regulation of plant-sulfur assimilation has not been reported. With an increasing understanding that soil microbes can activate growth and stress tolerance in plants via chemical signaling, the question arises as to whether such beneficial bacteria also regulate sulfur assimilation. Here we report a previously unidentified mechanism by which the growth-promoting rhizobacterium *Bacillus amyloliquefaciens* (GB03) transcriptionally activates genes responsible for sulfur assimilation, increasing sulfur uptake and accumulation in *Arabidopsis*. Transcripts encoding for sulfur-rich aliphatic and indolic glucosinolates are also GB03 induced. As a result, GB03-exposed plants with elevated glucosinolates exhibit greater protection against the generalist herbivore, *Spodoptera exigua* (beet armyworm, BAW). In contrast, a previously characterized glucosinolate mutant compromised in the production of both aliphatic and indolic glucosinolates is also compromised in terms of GB03-induced protection against insect herbivory. As with *in vitro* studies, soil-grown plants show enhanced glucosinolate accumulation and protection against BAW feeding with GB03 exposure. These results demonstrate the potential of microbes to enhance plant sulfur assimilation and emphasize the sophisticated integration of microbial signaling in plant defense.

## Introduction

Sulfur, a crucial element for plants, is ubiquitous in proteins, present in the antioxidant tripeptide glutathione, the Cys-rich peptides phytochelatins that function in heavy metals detoxification and thioredoxins that are the major disulfide reductases responsible for maintaining the reduced state of proteins inside cells (Arnér and Holmgren, 2000; Cobbett, 2000). Sulfur can also be present in chloroplastic membrane lipids as well as certain coenzymes/vitamins (Falk et al., 2007). Sulfur is taken up by plants as inorganic sulfate via sulfate transporters and incorporated into APS by ATPS (Mugford et al., 2009). APS is then sequentially reduced by APR and sulfite reductase to sulfite and sulfide, and subsequently incorporated into *O*-acetylserine to form the sulfur containing amino acid cysteine. APS can also be phosphorylated to PAPS by the action of APK. PAPS is the sulfate donor for the formation of sulfated metabolites including glucosinolates, select flavonoids, phytosulfokines, and certain hormones.

From an ecological context, sulfur metabolites function in plant defense against pathogens and herbivores (Falk et al., 2007). Defensin and thionin peptides are sulfur-containing antimicrobial defenses with widespread plant distribution (Broekaert et al., 1995), whereas anti-feedant glucosinolates are limited to the Brassicale order (Falk et al., 2007). *Brassica* crops including cabbage, broccoli, cauliflower (*Brassica oleracea*) and rapeseed (*B. napus*) as well as *Arabidopsis* are rich in glucosinolates. In addition to these amino acid derivatives functioning in plant defense, glucosinolates are a nutritional source of sulfur and possess cancer-preventive properties (Sønderby et al., 2010). Glucosinolates are classified based on their amino acid precursor with aliphatic glucosinolates derived from Met, Ala, Leu, Ile, or Val; indolic glucosinolates derived from Trp; and aromatic glucosinolates derived from Phe or Tyr (Kliebenstein et al., 2001).

With plant damage, glucosinolates are rapidly converted into an array of toxic derivatives that can obfuscate phytochemical analysis. Enzymatically generated glucosinolate derivatives including isothiocyanates, epithionitriles, nitriles, and thiocyanates are produced in proportion to the amount of leaf damage as well as the reaction time (Halkier and Gershenzon, 2006; Wittstock and Burow, 2010; Winde and Wittstock, 2011). Therefore quantifying the pool of original glucosinolates requires deactivating the myrosinase enzyme before glucosinolates are enzymatically converted (Koroleva et al., 2000; Andréasson and Jørgensen, 2003; Zhao et al., 2008; Winde and Wittstock, 2011).

In addition to constitutive glucosinolate accumulation serving in chemical defense against herbivore damage, soil-borne microbes such as mycorrhizal fungi and PGPR can induce plant defense responses (van Loon, 2007; van Wees et al., 2008; Yang et al., 2009; Pineda et al., 2010, 2012). PGPR are naturally occurring soil microorganisms that colonize roots and stimulate plant growth. Such bacteria are applied to a wide range of agricultural crops for the purpose of growth enhancement, including increased seed germination, plant weight, harvest yields, and disease resistance (Kloepper et al., 1980, 1991, 1999). *Bacillus subtilis* (GB03), recently re-named as *B. amyloliquefaciens* is a commercially available PGPR strain that can be introduced into the soil at the time of planting via seed coating since spores are stable over time (Choi et al., 2014). Unlike many plant-growth promoting rhizobacterial strains that activate plant growth by directly producing and releasing indole-3-acetic acid and/or gibberellins, GB03 emits a bouquet of volatile metabolites, devoid of classic phytohormones that are capable of triggering plant growth promotion (Ryu et al., 2003; Paré et al., 2005). These VOCs have been shown to activate differential expression of approximately 600 transcripts related to cell wall modifications, primary and secondary metabolism, stress responses, hormone regulation, and iron homeostasis (Ryu et al., 2003; Farag et al., 2006; Zhang et al., 2007). This *Arabidopsis* profiling of GB03-induced transcripts has resulted in a new paradigm for PGPR-mediated iron uptake. While some soil microbes are proposed to enhance iron mobility and uptake solely via production of bacterial siderophores (Neilands and Leong, 1986; Bar-Ness et al., 1992; Briat, 1992; Glick et al., 1999; Sharma et al., 2003), GB03 enhances *Arabidopsis* iron accumulation via activation of the plant’s own iron acquisition machinery including the iron uptake-related genes *FRO2* and *IRT1* that encode for ferric reductase and iron transport enzymes, respectively (Zhang et al., 2009). GB03 also transcriptionally regulates the Fe-deficiency-induced transcription factor 1 (*FIT1*) that is necessary and sufficient for ferric reductase and iron transporter induction (Zhang et al., 2009). More recently, an upstream iron acquisition-related transcription factor *MYB72* has been shown to be transcriptionally induced in *Arabidopsis* by bacterial VOCs with activation of the iron uptake-related genes *FIT1, FRO2*, and *IRT1* (Zamioudis et al., 2015).

The current study reports a novel mechanism in which the growth-promoting rhizobacterium *B. amyloliquefaciens* strain GB03 induces *Arabidopsis* sulfur assimilation and accumulation by inducing the plant’s own sulfur assimilation machinery. Moreover, the impact of GB03 in regulating primary and secondary sulfur metabolites to enhance plant defense against herbivory is examined.

## Materials and Methods

### Plant Material and Treatments

*Arabidopsis thaliana* seeds were surface sterilized and stratified for 2 days at 4°C in the absence of light. Seeds were planted in plastic Petri dishes (100 × 15 mm) containing a central partition (I-plates; Fisher Scientific), covered Magenta boxes (75 mm × 75 mm × 100 mm) or standard Petri dishes (150 mm × 15 mm), based on the specific experimental requirements. The bacterial culture is inoculated on the unplanted side of the partitioned plate, a glass vial (4 dr.) or a plastic plate (35 mm × 10 mm). All chambers contained half-strength MS solid media prepared according to Murashige and Skoog (1962) with 1.5% (w/v) sucrose and 0.8% (w/v) agar (except where noted otherwise). Plants were grown under a 14-/10-h light/dark cycle with metal halide and high pressure sodium lamps for a total light intensity of 200 μmol photons m^-2^ s^-1^; temperature was 21 ± 4°C and relative humidity 40 ± 10%. For plant growth, the media surface was oriented horizontally for I-plates and Magenta boxes and vertically for the larger plates with media agar increased to 1.5% (w/v).

*Bacillus amyloliquefaciens* (GB03) was streaked onto TSA plates and incubated at 28°C in the absence of light for 24 h. Cells were harvested in double distilled water (DDW) to yield 10^9^ CFU mL^-1^, as determined by optical density (OD_600_ = 0.7). Two days after seed germination, the bacterial suspension culture or DDW (25 μL for plates and 50 μL for Magenta boxes) was added to the non-plant portion of the chamber. Vials containing bacterial culture were replaced with fresh culture every 14 days.

For soil experiments, bacterial liquid cultures were mixed with sterile growing mix (Sunshine LC1 Mix; Sun Gro Horticulture, Canada) to a final PGPR concentration of 10^8^ – 10^9^ CFU/g soil. For water control, the bacterial suspension was replaced with sterile DDW. Seeds were sown in growing mix and fertilized weekly using 13:13:13 (N:P:K) fertilizer.

### Semi-quantitative RT-PCR

Plants were harvested 48- or 72-h after GB03 or water treatment. Total RNA was extracted using RNeasy plant mini kit (Qiagen, Valencia, CA, USA) with genomic DNA contamination excluded by DNase digestion. First strand cDNA was synthesized from 3-5 μg total RNA using MuMLV-RT (Fisher Scientific, Houston, TX, USA); primer sequences are shown (**Table 1**). The PCR reaction included an initial 3 min denaturation at 94°C, followed by 30 s at 94°C, 30 s at 54°C and 1 min at 72°C with 24–27 cycles (based on the optimized linear range for each pair of specific primers), a final 10 min extension at 72°C (T100 Thermal Cycler, Bio-Rad, Hercules, CA, USA). No-reverse-transcription controls were included with the PCR runs to confirm the absence of DNA contamination. Agarose gel electrophoresis were imaged with a Kodak Gel Logic 100 Imaging System (Fisher Scientific, Houston, TX, USA) and quantified using Image J 1.33u^1^ (National Institute of Health, USA). *TUB8* and *UBQ10* were employed for normalization as they were uniformly expressed in all tissues examined.

**Table 1:** Sequence of primers employed in the semi-quantitative RT-PCR analysis.

Gene Name	Primer Sequence (5′ to 3′)
ATPS1	Forward: GTTTCCTTCCCTTCCAAATC
	Reverse: GAGCCAGTTTCCAGCATTAG
ATPS3	Forward: GAATGAAACAGCACGAGAAG
	Reverse: CCAGGGCACATAAATCCATC
ATPS2	Forward: ATGCTGTTTTTGCGTTTCAG
	Reverse: ACGGCTTGTTGTTTTGCTTC
ATPS4	Forward: GCGTATGAGACAGCACGAG
	Reverse: AACCAACACCTTCCAACCAG
APR1	Forward: AGGTTTGGATGGTGGAGTTG
	Reverse: CATAAAGCACGACGATCCAAG
APR2	Forward: CGAATCTTGGGTTACTCGTG
	Reverse: CCTCCTTGATGTTCCCTTTG
APR3	Forward: GAGATGGTGGTGGGAAGATG
	Reverse: TGGAACGAGACTGGATGGTC
APK1	Forward: TCCACCACCGTGAGATATGA
	Reverse: ATCCGCAAAAAGCTTAGCAA
APK2	Forward: TGGCACGAGAGTTCGATATG
	Reverse: CAGCACTACCTCGCAATTCA
CYP79F1	Forward: TCCATGGCATCAATCACTCTAC
	Reverse: CATCAACATTCCAACCTCTCAA
SUR1	Forward: TCGTGCTGCTTACAGTGGTC
	Reverse: ACACAGGGGATGTCCTTGAG
FMO_GS-OX3_	Forward: ACCAATGTCCCGAGAGAAAGTA
	Reverse: GGAACGGAAATCTTCTCGTATG
UBQ10	Forward: CGATTACTCTTGAGGTGGAG
	Reverse: AGACCAAGTGAAGTGTGGAC
TUB8	Forward: CGTGGATCACAGCAATACAGAGCC
	Reverse: CCTCCTGCACTTCCACTTCGTCTTC

### Total Sulfur Determination

Shoots and roots were separated, oven-dried, pulverized, and converted to dry-ash by heating at 550°C for 3 h in the presence of Ag_2_O and NaHCO_3_ based on Kalra (1998). Dried tissue was then neutralized, diluted, and analysis via a barium chloride-gelatin turbidimetric assay (Tabatabai and Bremner, 1970). Standards were prepared as tissue material and diluted to a final concentration of 0–32 μg mL^-1^. Total sulfur was quantified spectrophotometrically at 420 nm based on a sulfur standard curve.

### ^35^SO_4_^-2^ Uptake Assay

For sulfate uptake measurements, plants were germinated on nylon mesh and grown vertically on media-containing plates with GB03 or water exposure for 11 days. Radio-labeling was initiated by submerging the roots into liquid media containing 37 MBq L^-1^ ^35^SO_4_^-2^ (Perkin–Elmer). After incubation for 30 min, roots were briefly rinsed with non-labeled medium to remove apoplastic radioactivity (modified protocol from Kataoka et al., 2004; Maruyama-Nakashita et al., 2004; Yoshimoto et al., 2007). After blotting, shoots and roots were weighed separately, transferred to scintillation vials and covered with 1 mL of 0.1 M HCl. Overnight-extracted samples were mixed with universal scintillation cocktail (4 mL; Fisher Scientific) and incorporated radioactivity measured by liquid scintillation counting.

### Cysteine Measurements

Whole plant tissue (0.1 g) was ground in liquid nitrogen and thiols were acid extracted using ice-chilled 0.1 N HCl (200 μL). The homogenate was centrifuged at 12,000 × *g* for 10 min at 4°C. Supernatant aliquots were neutralized with 200 mM HEPES (pH 12.4), reduced with dithiothreitol and sulfhydryl groups derivatized with monobromobimane (VWR). Separation, detection and quantification of fluorescent adducts was based on Schupp and Rennenberg (1988).

### Glucosinolates Analysis

Plants were shoot and root separated, frozen in liquid nitrogen and lyophilized. Tissue (20–50 mg) was extracted for 15 min in boiling aqueous 7.5 mM Pb(OAc)_2_/Ba(OAc)_2_ (4 mL) with 0.57 μmol internal standard (sinigrin, Sigma–Aldrich) based on Reintanz et al. (2001). At room temperature, samples were gently shaken for 30 min, centrifuged at 4000 × *g* for 10 min and the supernatant was loaded on DEAE Sephadex A-25 column (120 mg, Sigma–Aldrich). Resin was rinsed with aqueous methanol (67%) and water and subsequently incubated with 50 μL sulfatase solution overnight (Graser et al., 2000). The resulting desulfoglucosinolates were eluted with 60% aqueous methanol (800 μL) and water (800 μL). The pooled extract was evaporated to dryness *in vacuo* and the residue was dissolved in HPLC-grade water (100 μL).

Desulfoglucosinolates were separated by HPLC on a Dionex Ultimate 3000 UHPLC system equipped with auto-sampler, column oven, and diode array detector. A C_18_ reversed phase column (Acclaim 120 mm × 3.0 mm, 150 mm × 3.0 mm i.d., 3-μm particle size) was run with a 400 μL/min flow rate at 25°C; the injection volume was 10 μL. Elution was performed with a gradient (solvent A water; B acetonitrile) of 1.5 to 5% solvent B (6 min), 5 to 7% solvent B (2 min), 7 to 21% solvent B (10 min), 21 to 29% solvent B (5 min), and 29 to 57% solvent B (14 min), followed by a cleaning cycle (57 to 93% solvent B for 3 min, 6 min of hold, 93 to 1.5% solvent B for 3 min with a 5 min hold). Compounds were monitored at 229 nm.

Desulfoglucosinolates were identified by HPLC-PDA-MS based on method of Kusznierewicz et al. (2013). Samples were analyzed on a LCQ Fleet HPLC system equipped with PAL autosampler, Surveyor PDA detector, and Surveyor MS pump using an Alltima C_18_ reversed phase column (250 mm × 2.1 mm i.d., 5-μm particle size) with a 200 μL/min flow rate. The injection volume was 10 μL. Elution was performed with a gradient (solvent A water/0.1% formic acid; B acetonitrile/0.1% formic acid) of 1.5% solvent B (3 min) 1.5 to 13% solvent B (15 min), 13 to 33% solvent B (12 min), 33 to 57% solvent B (7 min), followed by a cleaning cycle (57 to 93% solvent B for 3 min, 6 min of hold, 93 to 1.5% solvent B for 3 min with a 5 min hold). Compounds were monitored by PDA at 229 nm, then subsequently by ESI-MS (LCQ Fleet Ion Trap MS) operated in positive ion mode, an acquisition time of 40 min with scanning from *m*/*z* 150 to 800 amu.

Previously reported desulfoglucosinolates were identified by MS via characteristic [M+H]^+^ and [M+Na]^+^ peaks except for 3-methylsulfinylpropyl glucosinolate (3MSOP) which could not be identified because of poor resolution. Positional isomers 4MOI3M and 1MOI3M with equivalent masses were differentiated based on retention time comparisons with literature values (Reintanz et al., 2001). Glucosinolates were quantified based on response factors established for individual desulfoglucosinolates relative to the internal standard at 229 nm (Brown et al., 2003).

### Herbivore Feeding

*Spodoptera exigua* (BAW) eggs were purchased from Benzon research (Carlisle, PA, USA). After hatching, neonate larvae were transferred to feed on artificial media for 6 days with a transfer to fresh media every 2–3 days. Since an acclimation period is required whenever larvae are transferred from one diet to another, 1 day before the experiment, third-instar larvae were transferred to feed on non-experimental wild-type *Arabidopsis* plants (Mewis et al., 2005). After this pre-feeding, larvae of the same developmental stage were weighted and transferred to 29-day-old GB03- or water-treated plants (one larva/plant); the initial average weight of larvae was recorded for both GB03 and water treatments. Shoot biomass was recorded after 56 h of feeding. Additional GB03-treated and untreated plants were reserved to serve as undamaged controls. Plants were harvested, rinsed, and weighted. Milligrams eaten per plant were calculated based on the weight difference between BAW eaten and uneaten plants. The quadruple glucosinolate knock-out mutant (*myb28 myb29 cyp79b2 cyp79b3*) was treated the same as Col-0.

For soil experiments, herbivore weights were collected. Neonate larvae were transferred to 28-day-old GB03- or water-treated plants with a transfer to fresh plants every 2–3 days. Larvae weight was measured at 7 and 9 days after feeding.

### Statistical Analysis

For herbivore feeding experiments, statistical analyses were performed using R software^2^. First, a Levene’s test was performed to check the homogeneity of variance (Levene, 1960); homogeneous variance was achieved after transforming the data into the corresponding square root. Then, two-way ANOVAs were performed separately for wild-type and knock-out mutant lines. Tukey’s method was used to do pair-wise comparisons of means and an “lsmeans” package was used for means’ grouping. For all other experiments, pair-wise comparison of means was performed using Excel 2007 with significant difference between treatments was based on Student’s *t*-test at *P*-values ≤ 0.05. The number of biological replicates is shown in each figure legend with minimum of three replicates.

## Results

### Elevated Sulfate Assimilation with GB03 Exposure

**FIGURE 1:**
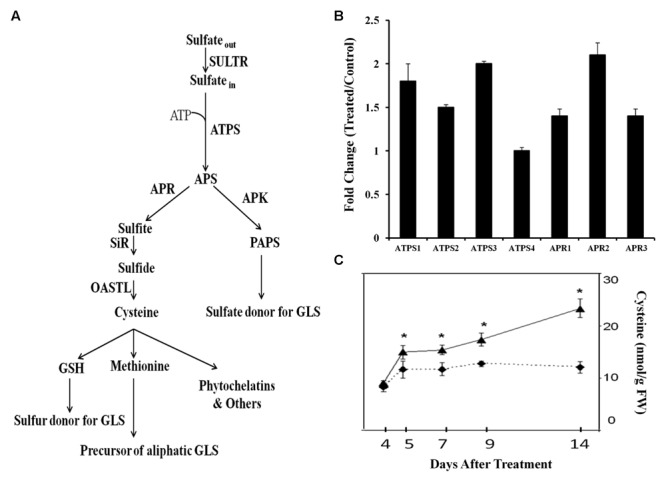
**Transcriptional regulation of *Arabidopsis* sulfur assimilation genes by GB03.** Depicted sulfate assimilation pathway adapted from Mugford et al. (2009) **(A)**. Semi-quantitative RT-PCR analysis of whole-plant sulfur assimilation gene expression at 72 h post GB03 treatment **(B)**; data are the averages of three biological replicates with error bars representing standard error. The amino acid cysteine increases with GB03 treatment (solid line) relative to the water controls (dashed lines; **C**); an asterisk (^∗^) indicate statistically significant difference between treatments (*t*-test, *P*-value ≤ 0.05, *n* = 6, mean ± SE). Sulfur assimilation pathway includes SULTR, sulfate transporter; ATPS, ATP sulfurlyase; APS, adenosine 5′-phosphosulfate; APR, APS reductase; SiR, sulfite reductase; OASTL; *O*-acetylserine (thiol) lyase; GSH, glutathione; APK, APS kinase; PAPS, 3′-phosphoadenosine 5′-phosphosulfate.

The sulfate assimilation pathway with previously identified genes is depicted in **Figure 1A**. Mining whole-plant microarray data of GB03-exposed *Arabidopsis* seedlings identified sulfate-assimilation gene induction for *ATPS* and *APR*. Of the four ATPS and the three APR isozymes present, *ATPS1* and *ATPS3* as well as *APR1* and *APR2* were found to be induced at 72 h post GB03 exposure (Supplementary Figure 1). RT-PCR analysis confirmed GB03 induction for *ATPS1, ATPS2*, and *ATPS3* and all *APR* genes (**Figure 1B**). Another branch of sulfur assimilation involves APS conversion to PAPS by APK. There are four functional APK isoforms in *Arabidopsis*, among them APK1 and APK2 are the most active isoforms (Mugford et al., 2009). From the microarray data, both *APK1* and *APK2* are GB03 up regulated relative to controls (Supplementary Figure 1). *APK* transcript induction confirmation via RT-PCR analysis showed GB03 induction only in shoots (*APK1*, 1.3 ± 0.08; *APK2*, 1.5 ± 0.05). In addition, the amino acid cysteine, a precursor of many organic sulfur metabolites increased 28 ± 11, 32 ± 8, 37 ± 10, and 93 ± 15 % with GB03 exposure at 5, 7, 9, and 14 days, respectively (**Figure 1C**).

### GB03 Enhances Sulfur Accumulation and Uptake

**FIGURE 2:**
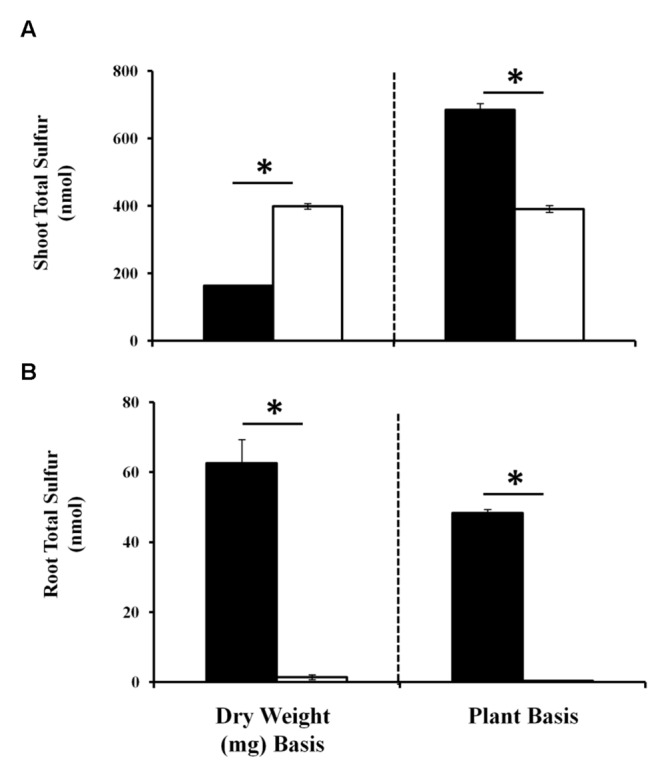
**Sulfur accumulation in *Arabidopsis* with GB03 exposure.** Shoot **(A)** and root **(B)** sulfur accumulation in 13-day-old plants that are GB03- (black bars) or water-treated (white bars) on a dry-weight and per-plant basis. Root sulfur values for water-treated plants are 1.36 ± 0.68 nmol/mg DW and 0.4 ± 0.05 nmol/plant, although values are not perceivable in the figure. An asterisk (^∗^) indicates statistically significant difference between treatments (*t*-test, *P*-value ≤ 0.05, *n =* 4, mean ± SE).

**FIGURE 3:**
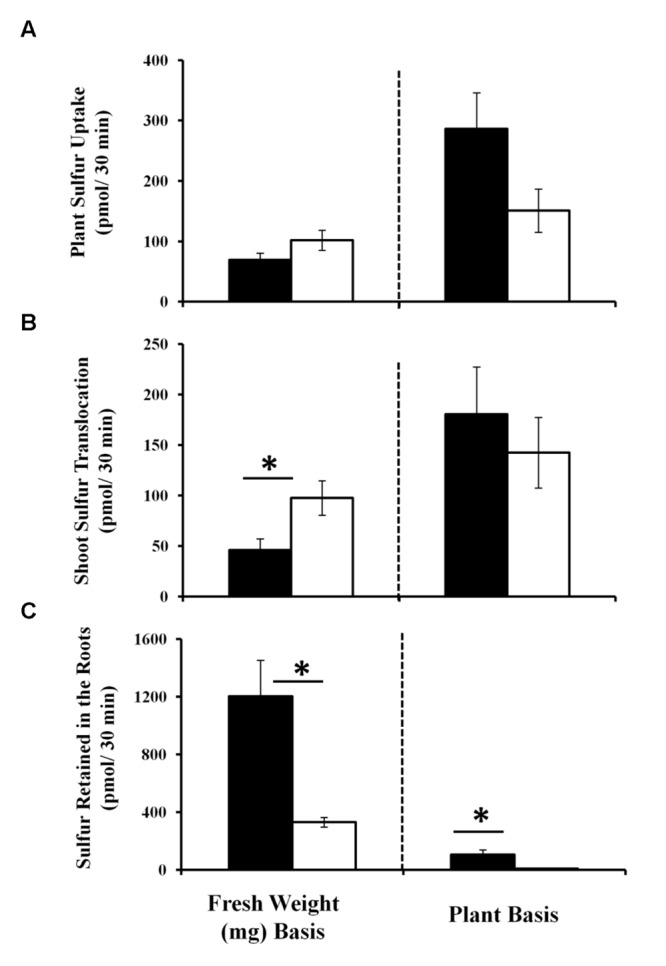
**Sulfur uptake and translocation monitored by radioactive sulfate (^35^SO_4_^-2^ 30 min pulse) in *Arabidopsis* with GB03 exposure.** Whole plant sulfur uptake **(A)**, shoot sulfur translocation **(B)** and sulfur root retention **(C)** is shown in 13-day-old plants that are GB03- (black bars) or water-treated (white bars) on a fresh-weight and per-plant basis. The value of sulfur retained in the roots for water-treated plants is 8.6 ± 0.98 pmol/30 min/plant, although values are not perceivable in the figure. An asterisk (^∗^) indicates statistically significant difference between treatments (*t*-test, *P*-value ≤ 0.05, *n ≥* 4, mean ± SE).

As sulfate assimilation and reduction genes were GB03 induced, sulfur accumulation was examined. While total sulfur accumulation per tissue weight decreased *ca.* twofold in shoots 11 days post GB03 exposure, shoot sulfur accumulation per plant increased *ca.* 75% (**Figure 2A**). In roots, increases of *ca.* 50-fold and *ca*. 100-fold on a dry-weight and per-plant basis, respectively, were observed (**Figure 2B**). To better characterize the process of inducible sulfur metabolism, plant sulfur movement was monitored with radioactive sulfate (^35^SO_4_^-2^) to examine sulfur uptake and translocation. Although there was a *ca.* 30% reduction in total sulfur uptake per tissue weight, GB03 exposure enhanced total sulfur uptake per plant by *ca.* twofold, relative to untreated controls, within 30 min of radio-labeling (**Figure 3A**). Shoot sulfur translocation per tissue weight was *ca.* twofold less with GB03 treatment; however, similar translocation rate per plant was observed for both GB03 and controls (**Figure 3B**). And in roots, sulfur uptake and retention was higher with GB03 exposure on both a tissue weight and per-plant basis (**Figure 3C**).

Since select bacterial volatiles such as 2,3-butandiol have been previously shown to induce growth promotion and induced systemic resistance in *Arabidopsis* (Ryu et al., 2003, 2004), an array of 2,3-butandiol concentrations were assayed to examine for enhanced sulfur accumulation albeit no sulfur-associated changes were detected (data not shown). Similarly, collected bacterial volatiles re-introduced to plants also did not enhance sulfur accumulation.

### GB03 Induces Glucosinolate Biosynthetic Transcripts

**FIGURE 4:**
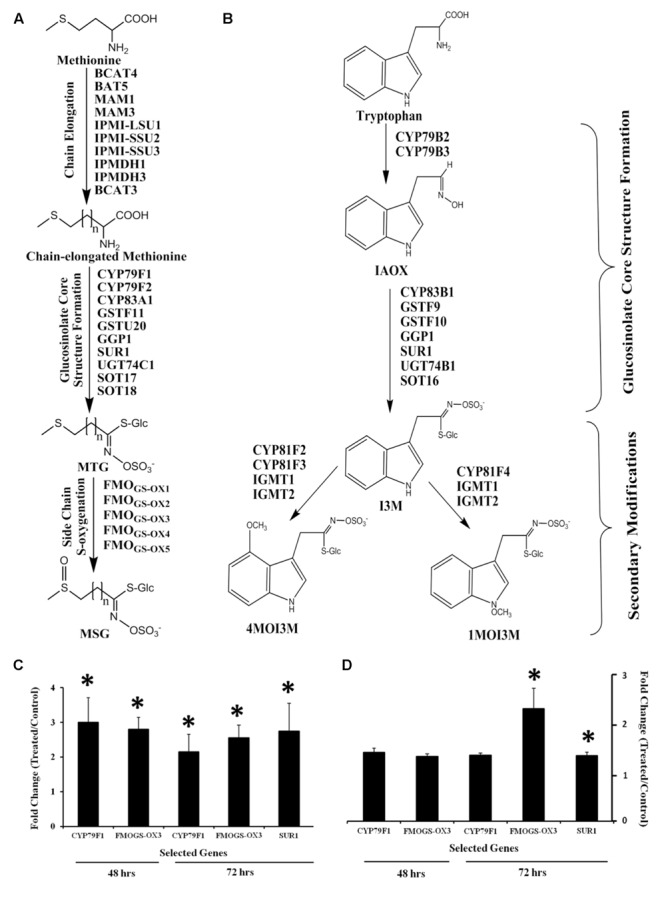
**Glucosinolate biosynthesis transcriptional regulation in *Arabidopsis* by GB03.** Depicted aliphatic **(A)** and indolic **(B)** glucosinolate biosynthetic pathways adapted from Sønderby et al. (2010). Semi-quantitative RT-PCR analysis of *CYP79F1* and *FMO_GS-OX3_* gene expression in both shoots **(C)** and roots **(D)** at 48 and 72 h, and *SUR1* at 72 h post GB03 exposure; an asterisk (*) indicates statistically significant difference between treatments (*t*-test, *P*-value ≤ 0.05, *n* = 3, mean ± SE).

The aliphatic and indolic glucosinolate biosynthetic pathways with previously identified genes is depicted in **Figures 4A,B**, respectively. Mining microarray data for transcripts encoding glucosinolate biosynthesis revealed that the majority of aliphatic pathway genes are GB03 induced (Supplementary Figure 2A). For indolic glucosinolate biosynthesis, microarray data showed transcript induction limited to *GSTF9, SUR1, UGT74B1*, and *SOT16* (Supplementary Figure 2B). GSTF9 is a GST which is responsible for the conjugation of the activated aldoximes to the sulfur donor glutathione, where the resulting *S*-alkylthiohydroximates are converted to thiohydroximates by a carbon-sulfur lyase, SUR1. Thiohydroximates are in turn *S*-glucosylated by the glucosyltransferases UGT74B1 to form desulfoglucosinolates. Finally, desulfoglucosinolates are sulfated to the corresponding glucosinolates by the sulfotransferase SOT16. Monitoring select shoot and root transcripts separately by RT-PCR confirmed gene induction with *CYP79F1* induction in shoots *ca*. threefold, while root induction was *ca.* 30% (**Figures 4C,D**). CYP79F1 catalyzes the first committed step in biosynthesis of the aliphatic glucosinolate core structure that involves conversion of amino acids to corresponding aldoximes (a rate-limiting step in glucosinolates biosynthesis; Mikkelsen and Halkier, 2003). *FMO_GS-OX3_*, a gene that encodes one of the five flavin monooxygenases responsible for *S*-oxygenation of aliphatic glucosinolates resulting in conversion of MTG to MSG (Sønderby et al., 2010) was induced in shoots within 48 h while root induction was delayed to 72 h (**Figures 4C,D**). *SUR1* gene expression was induced *ca*. threefold in shoots (**Figures 4C,D**). To link transcriptional regulation with downstream glucosinolate accumulation, qualitative and quantitative glucosinolate analysis was performed.

### GB03 Induces Glucosinolate Accumulation

**FIGURE 5:**
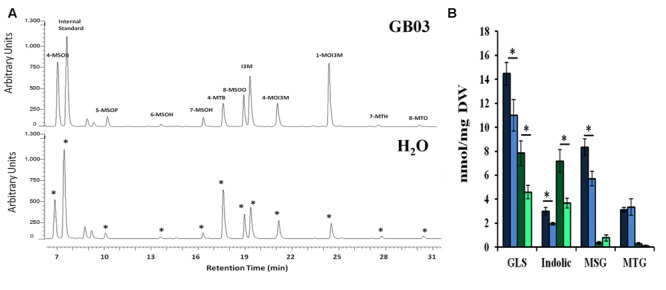
**Glucosinolate accumulation in *Arabidopsis* by GB03.** Representative UHPLC-PDA chromatogram showing shoot glucosinolate profile in both GB03- and water-treated 30-day-old plants **(A)**; an asterisk (^∗^) indicates peak alignment between the lower and upper chromatograms. GB03 induces total glucosinolate (GLS) accumulation in shoots (dark blue-GB03 versus light blue bar-H_2_O control) and roots (dark green-GB03 versus light green bar-H_2_O control) in 30-day-old plants **(B)**. The aliphatic MSG and MTG as well as indolic glucosinolates are shown; an asterisk (^∗^) indicates statistically significant difference between treatments (*t*-test, *P*-value ≤ 0.05, *n* = 6, mean ± SE). Internal standard, sinigrin; 4-MSOB, 4-methylsulfinylbutyl glucosinolate; 5-MSOP, 5-methylsulfinylpentyl glucosinolate; 6-MSOH, 6-methylsulfinylhexyl glucosinolate; 7-MSOH, 7-methylsulfinylheptyl glucosinolate; 4-MTB, 4-methylthiobutyl glucosinolate; 8-MSOO, 8-methylsulfinyloctyl glucosinolate; I3M, indol-3-ylmethyl glucosinolate; 4MOI3M, 4-methoxyindol-3-ylmethyl glucosinolate; 1MOI3M, 1-methoxyindol-3-ylmethyl glucosinolate; 7-MTH, 7-methylthioheptyl glucosinolate; and 8-MTO, 8-methylthiooctyl glucosinolate.

Desulfoglucosinolates were separated by HPLC based on relative polarity, with MSG eluting first in increasing order of their side-chain length, followed by indolic glucosinolates; long chain MTGs eluted last off the column (**Figure 5A**). GB03 exposure resulted in *ca.* 33 and 70% greater glucosinolate accumulation in shoots and roots, respectively (**Figure 5B**). Specifically, GB03 increased indolic glucosinolates in shoots (55%) and roots (twofold) while MSGs were induced in shoots by 45%. MTG accumulation differences with regard to tissue or GB03 treatment was not observed. In shoots, I3M was the most GB03 induced indolic glucosinolate (68%; **Table 2**); while among MSG, there was a 35, 37, 69, 73, and 65% GB03 induction of 4-MSOB, 5-MSOP, 6-MSOH, 7-MSOH, and 8-MSOO, respectively. For roots, the most abundant glucosinolate, 1MOI3M, increased threefold with no other statistically significant accumulation changes. Glucosinolate accumulation was not induced with plant exposure to 2,3-butandiol or collected bacterial volatiles (data not shown).

**Table 2:** GB03-induced glucosinolate (nmol/mg DW) accumulation with 30-day-old plants.

Systematic Name	Common Name	Shoot			Root		
		GB03	H_2_O	Fold Change	GB03	H_2_O	Fold Change
4-MSOB	Glucoraphanin	**4.43 ± 0.40**	**3.28 ± 0.26**	1.35	Nd	Nd	–
5-MSOP	Glucoalyssin	**0.657 ± 0.061**	**0.48 ± 0.038**	1.37	Nd	Nd	–
6-MSOH	Glucohesperin	**0.147 ± 0.014**	**0.087 ± 0.012**	1.69	Nd	Nd	–
7-MSOH	Glucoibarin	**0.623 ± 0.057**	**0.359 ± 0.051**	1.73	0.12 ± 0.027	0.32 ± 0.11	0.38
4-MTB	Glucoerucin	2.74 ± 0.19	2.98 ± 0.64	0.92	Nd	Nd	–
8-MSOO	Glucohirsutin	**2.48 ± 0.22**	**1.50 ± 0.277**	1.65	0.25 ± 0.088	0.45 ± 0.13	0.56
I3M	Glucobrassicin	**1.61 ± 0.12**	**0.958 ± 0.083**	1.68	0.16 ± 0.033	0.30 ± .066	0.53
4MOI3M	4-Methoxygluco-brassicin	**0.825 ± 0.046**	**0.657 ± 0.025**	1.25	1.16 ± 0.21	1.28 ± 0.36	0.90
1MOI3M	Neoglucobrassicin	0.56 ± 0.178	0.32 ± 0.048	1.71	**5.85 ± 0.74**	**2.10 ± 0.29**	2.78
7-MTH	–	0.17 ± 0.0062	0.16 ± .022	1.10	0.086 ± .028	0.036 ± .017	2.37
8-MTO	–	0.20 ± 0.0064	0.20 ± 0.029	0.97	0.198 ± 0.069	0.083 ± .024	2.37

*Bold values indicate statistically significant difference between treatments (*t*-test, *P-*value ≤ 0.05, *n* = 6, mean ± SE).*

### GB03 Induces Plant Biomass Protection with Herbivory

**FIGURE 6:**
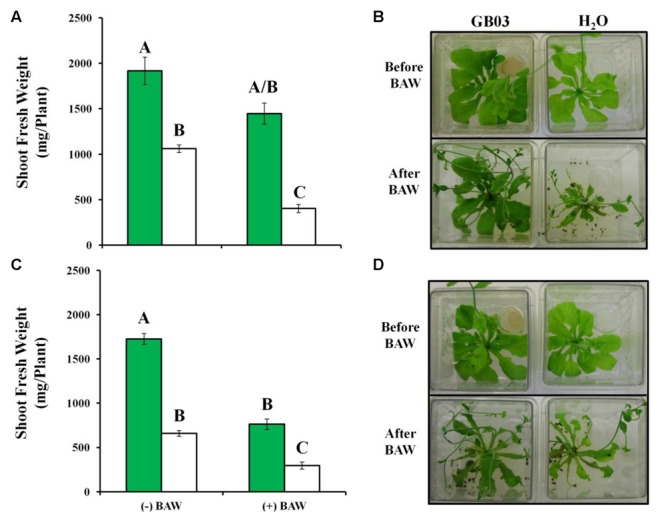
***Arabidopsis* protection against the generalist herbivore *Spodoptera exigua* (BAW) by GB03.** Twenty nine-day-old wild-type **(A,B)** and glucosinolate quadruple *myb28 myb29 cyp79b2 cyp79b3* mutant plants **(C,D)** without (–BAW) and with (+BAW) larval feeding (third instar) for 56 h. GB03-treated (green bars) and H_2_O-control plants (white bars) are shown. Data were analyzed by two-way ANOVA; different letters indicate statistically significant differences between treatments (Tukey’s test, *P*-value ≤ 0.01, *n* ≥ 11, mean ± SE). Representative plant images **(B,D)** are shown.

GB03-treated plants were approximately twice the weight of water controls (**Figure 6A**). For feeding experiments, third instar larvae were pre-fed for 1 day on non-exposed *Arabidopsis* seedlings and initial BAW weight was monitored for larvae that were to feed on GB03 (46.39 ± 2.48) or water (46.48 ± 1.68) treated plants to exclude the effect of larvae weight and developmental stage variation on larval feeding. In addition, vials containing GB03 were removed from plant chambers before introducing BAW to avoid any direct interactions between bacterial volatiles and larvae as well as to avoid unequal PGPR-mediated growth for plants without BAW. With larval feeding, GB03-treated plants lost 24% shoot weight while controls lost 62% weight within 56 h of BAW feeding; plant tissue eaten per plant was 469 ± 54 and 658 ± 20 mg for GB03 and water treatments, respectively (*t*-test, *P* = 0.004, *n* = 11).

### GB03 Induces Glucosinolate-Dependant Plant Biomass Protection with Herbivory

For plants without herbivory, similar GB03-induced growth promotion was observed for both the Col-0 and a glucosinolate knock-out line (**Figures 6A–D**) compromised in both aliphatic and indolic glucosinolate production, *myb28 myb29 cyp79b2 cyp79b3* (Müller et al., 2010). With larval feeding on the knock-out line, shoot weight loss of 55% was observed for both GB03 and water treated plants; tissue consumed per plant was 962 ± 13 and 364 ± 11 mg for GB03 and water treatments, respectively (*t*-test, *P* = 1.89*E* – 20, *n* ≥ 11).

### GB03 Induces Plant Biomass Protection with Herbivory *In Vivo*

**FIGURE 7:**
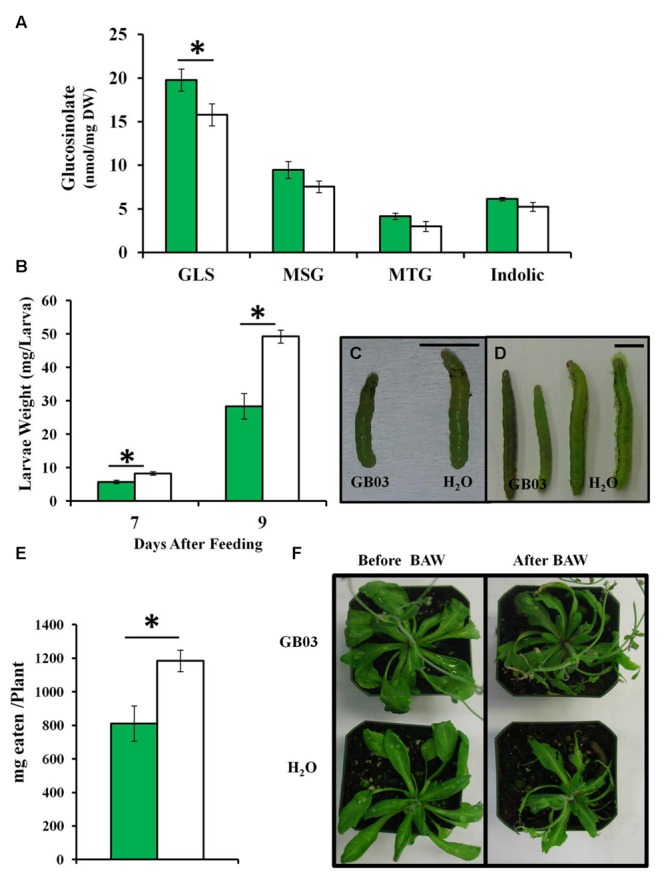
**Glucosinolate accumulation, BAW weight and leaf damage in soil-grown *Arabidopsis* with GB03 treatment.** Shoot total GLS accumulation in 35-day-old plants that are GB03- (green bars) or water-treated (white bars; *n* = 5; **A**). The aliphatic MSG and MTG as well as indolic glucosinolates are shown. Forth instar larvae were weighed 7 (*n* ≥ 18) and 9 days (*n* ≥ 11) after feeding on 28-day-old plants **(B)**; representative images at 7 **(C)** and 9 days **(D)** are shown with the bar scale representing 0.5 cm. Milligrams eaten per plant after 3 days of larvae feeding on 37-day-old plants (*n* = 10; **E**). Representative plant images are shown **(F)**. An asterisk (^∗^) indicates statistically significant difference between treatments (*t*-test, *P*-value ≤ 0.05, mean ± SE).

GB03-treated soil-grown Col-0 plants accumulate *ca*. 25% higher levels of glucosinolates compared to water controls in shoots for 35-day-old plants (**Figure 7A**). In addition, larval weight was lower when fed on GB03-treated plants for 7 and 9 days compared with controls (**Figures 7B–D**). Moreover, in the presence BAW, plant tissue eaten per plant was less for GB03-treated plants versus water controls (**Figures 7E,F**). A model for GB03-conferred protection against herbivory is proposed (Supplementary Figure 3).

## Discussion

Several responses are induced in *Arabidopsis* by the PGPR strain GB03 including enhanced photosynthetic efficiency (Zhang et al., 2008), increased iron assimilation (Zhang et al., 2009) and elevated reproductive success (Xie et al., 2009), however, the ability of PGPR to induce sulfur assimilation via established mechanisms operational in plants has not been previously reported. Here is described that sulfur assimilation and glucosinolate biosynthetic genes are transcriptionally up regulated with GB03 exposure in *Arabidopsis*, from literature extracted microarray data (Zhang et al., 2007) and RT-PCR analysis. At the metabolite level, enhanced sulfate uptake along with elevated total sulfur, cysteine and sulfated aliphatic/indolic glucosinolate accumulation is observed. GB03-exposed plants also exhibit greater protection against the generalist herbivore BAW, while enhanced protection is compromised in a glucosinolate quadruple knockout line. Consistent with *in vitro* studies, GB03 enhanced glucosinolate accumulation and protection against larval feeding with soil-grown plants.

The PGPR strain *Bacillus* sp. B55 has previously been shown to promote tobacco growth by enhancing sulfur nutrition via uptake of sulfur volatiles including the major bacterial volatile component, DMDS (Meldau et al., 2013). B55 DMDS sulfur uptake observed in tobacco is subsequently incorporated into plant proteins and accompanied by reduced gene expression involved in sulfur assimilation, Met biosynthesis and sulfur recycling. In contrast, GB03 VOCs are low in sulfur emissions (Farag et al., 2006) and up-regulate genes that mediate sulfur assimilation. Since the volatilome has only been chemically characterized for GB03 (Farag et al., 2006), a unified mechanism for chemical incorporation and/or signaling inducing sulfur metabolism by GB03 and B55a is not possible. Moreover, different sulfur demands between glucosinolate-rich cruciferous plants such as *Arabidopsis* and glucosinolate-deficient tobacco (Falk et al., 2007), also prevents direct comparisons between the two sulfur induction studies.

In *Arabidopsis*, sulfate is taken up by roots and although root plastids contain the enzymatic machinery for sulfate reduction, sulfate conversion to sulfide and subsequent incorporation into cysteine predominantly takes place in shoot chloroplasts (Davidian and Kopriva, 2010). GB03 induces several *Arabidopsis* sulfate reduction genes including the key drivers of sulfate assimilation ATPS1 and APR2 (Loudet et al., 2007; Koprivova et al., 2013). While low-level gene activation does not constitute transcriptional regulation, the comprehensive induction of sulfate assimilation genes observed with GB03 exposure is consistent with coordinated transcriptional control. GB03-induced sulfur assimilation correlates with enhanced sulfur uptake and accumulation in roots. In shoots, sulfur uptake and accumulation per tissue weight is lower with GB03 exposure (**Figures 2** and **3**) which may be in part due to a dilution of plant sulfur with enhanced growth induced by GB03. On a whole-plant basis, sulfur uptake and accumulation is uniformly higher with GB03 treatment: 286.37 ± 59 versus 151 ± 35 pmol/30 min sulfur uptake and 732.6 ± 20 versus 391.36 ± 10 nmol accumulation for GB03 versus water treatments, respectively. Sulfur content in for *in vitro* grown *Arabidopsis* with and without GB03 exposure are consistent with published ICP-MS sulfur quantification under the same experimental conditions (Kwon et al., 2010). In addition to enhancing sulfur accumulation, GB03 has been previously shown to enhance *Arabidopsis* iron and copper accumulation (Zhang et al., 2009; Kwon et al., 2010), suggesting that this may be a coordinated effort by bacteria to increase plant growth by effectively enhancing the accumulation of essential elements.

GB03-induced sulfur assimilation enhances accumulation of cysteine, the precursor of methionine, GSH and subsequently select glucosinolates. Methionine is the main substrate for aliphatic glucosinolates (Ravanel et al., 1998) while the active sulfate donor for glucosinolate biosynthesis is PAPS, a phosphorylated derivative of APS produced by APK (Mugford et al., 2009; Sønderby et al., 2010). The crucial role of APK1 and APK2 in glucosinolate biosynthesis has previously been established using an *apk1 apk2* double mutant which resulted in an 80% glucosinolate reduction and a concomitant increase in desulfoglucosinolates (Mugford et al., 2009). The transfer of the sulfate group from PAPS to the free hydroxyl group of desulfoglucosinolates is catalyzed by SOTs (Sønderby et al., 2010). The parallel transcriptional up-regulation of *APKs* and *SOTs* suggest a coordinated regulation of the sulfate donor formation and the sulfate transfer reaction by GB03.

The induction of glucosinolate accumulation in response to herbivore attack has been extensively studied (Mewis et al., 2006; Hopkins et al., 2009), however, much less is known with regard to microbial glucosinolate induction (van de Mortel et al., 2012). With the root-colonizing *Pseudomonas fluorescens* strain SS101 (*Pf.*SS101), the phytoalexin camalexin and glucosinolates were correlated with induced systemic resistance in *Arabidopsis* against several bacterial pathogens, including *Pseudomonas syringae* pv *tomato* (*Pst*). In addition, herbivore mortality rate was greater with BAW feeding on *Pf.*SS101-root-colonized *Arabidopsis* and mortality-rate differences with SS101 root inoculation were lost when an indolic glucosinolate deficient line was assayed (van de Mortel et al., 2012). In this current report, inducible sulfur assimilation and/or partitioning is/are linked with elevated endogenous glucosinolates, foliar plant biomass with herbivory and larval weight. By monitoring enhanced plant protection against BAW feeding by bacterial volatiles albeit devoid of direct plant–bacteria contact, induced plant defense responses independent of potential confounding bacterial anti-feedant effects can be identified. Without BAW larvae present, GB03 induced plant growth in both the wild-type and glucosinolate mutant line (**Figure 6**), indicating that glucosinolates play no role in GB03-triggered growth promotion. However, greater GB03-induced growth promotion in the mutant line compared to Col-0 (**Figure 6C**) may be in part due to additional energy available for growth promotion without glucosinolate biosynthesis operative. With BAW herbivory, GB03-treated Col-0 plants lost less shoot weight than water controls (**Figures 6A,B**), indicating GB03-induction of plant defense(s). With such GB03 plant protection against larval feeding compromised in the glucosinolate mutant line (**Figures 6C,D**), a causal relationship is established between GB03-enhanced glucosinolate accumulation and conferred plant protection. Interestingly, in the mutant line without glucosinolate defenses present, larval-consumed plant tissue per plant was greater with versus without GB03 exposure; tissue consumed per plant was 962 ± 13 and 364 ± 11 mg for GB03 versus water treatments, respectively. Future experiments will examine if GB03-induced plants contain greater amounts of young leaves that have yet to accumulate non-glucosinolate based chemical defenses or if such plants dilute non-inducible chemical defenses making the GB03-induced glucosinolate mutant line more palatable for feeding larvae.

Soil-grown GB03-treated plants exhibited enhanced glucosinolate accumulation and plant protection against BAW is consistent with I-plate experiments; however, elicitation differences limit direct comparisons between *in vitro* and *in vivo* systems. For example, chemical signaling is confined to bacterial VOCs *in vitro* while non-volatile metabolites can also serve as potential signaling molecules in the *in vivo* soil system. Moreover, although the soil is sterilized before planting and bacterial inoculation, the non-sterile environment in which soil-grown plants are exposed is conducive to bacterial proliferate of leaves and roots by other bacterial strains besides GB03. Down-stream signaling pathways can also be differentially regulated in media and soil systems. For example, ethylene signaling is operative with *in vivo* PGPR signaling but not with *in vitro* growth promotion (Ryu et al., 2005). Future studies will examine several mutant lines to elucidate which of the different plant signaling pathways are involved in eliciting enhanced sulfur metabolism and protection against herbivores by GB03 both *in vitro* and *in vivo*. Moreover, since it has been widely recognized that the plant hormone jasmonic acid (JA) plays a crucial role in plant defense against pathogens and herbivores as well as in glucosinolate accumulation (van Dam et al., 2004; van Dam and Oomen, 2008), JA mutant lines will be assayed.

Since the growth promotion signal 2,3-butandiol (Ryu et al., 2003, 2004) as well as collected GB03 VOCs re-introduced to plants do not exhibit enhanced sulfur assimilation or glucosinolate accumulation a more effective absorbent may be necessary to trap biologically active bacterial volatiles. Alternatively, as the genome sequence of GB03 has been recently identified (Choi et al., 2014), testing different GB03 mutant lines could help deciphering the effect of different VOCs products on inducing sulfur metabolism.

Glucosinolate accumulation differs between shoots and roots. Without GB03 exposure, total glucosinolates are higher in shoots with aliphatic glucosinolates being the most abundant compared with roots. The subclass of indolic glucosinolates accumulates predominately in roots as has been reported previously in *Arabidopsis* (Brown et al., 2003) as well as in other *Brassica* species (Rosa, 1997; Kirkegaard and Sarwar, 1998). An absence of detectable short chain aliphatic glucosinolates in roots (**Table 2**) is consistent with recent findings where rosette leaves are the major source and storage site for short chain aliphatic glucosinolates (Andersen et al., 2013). GB03 induces a 33 and 70% increase in total glucosinolate content in shoots and roots, respectively. Although tissue perception of bacterial VOCs is unknown, GB03-induced increase in glucosinolates is higher in roots, suggesting that GB03 may have initially been recognized as a pathogen with glucosinolates potentially induced as a defense mechanism. In fact, *Arabidopsis* indolic glucosinolates are pathogen-induced by *Erwinia carotovora* (Brader et al., 2001).

In agriculture, in addition to generating defense-rich plants, sulfur-rich cruciferous crops such as canola (*Brassica napus* L. cv) require uniform sulfur uptake independent of soil sulfur content (Scherer, 2001). Other plant specific soil bacteria, active in triggering canola growth promotion have been examined, although their role in regulating sulfur assimilation has yet to be characterized (Kloepper et al., 1988; Bertrand et al., 2001). Commercial canola inoculants have been developed that oxidize elemental sulfur to the sulfate form that is more readily taken up by plants. Such bacterial inoculants are agriculturally relevant since elemental sulfur, an industrial by-product, is economically viable for regenerating sulfur deficient soils. Here, GB03 transcriptionally induces sulfate assimilation and coordinates this process with enhanced sulfate uptake as well as elevated sulfur, cysteine, and glucosinolate accumulation. In addition to the role of glucosinolates in plant defense, select sulfur metabolites possess cancer-preventive properties. For humans, isothiocyanates derived from the hydrolysis of MSG are potent cancer-preventive agents (Hansen et al., 2007; Li et al., 2008). The cancer-preventive properties of MSG have been targeted for elevated production by plant breeders of cruciferous crops (Li et al., 2008) and here they are shown to be selectively induced by GB03, relative to other aliphatic glucosinolates.

## Author Contributions

MA designed the project, performed experiments, collected data, analyzed results, and wrote up the study; RN performed experiments, collected data, and analyzed results; XX designed the project, performed experiments, collected data, and analyzed data; YS collected data; KS collected data; J-LZ designed the project and analyzed the results; and PP designed the project, analyzed results, and wrote up the study.

## Conflict of Interest Statement

The authors declare that the research was conducted in the absence of any commercial or financial relationships that could be construed as a potential conflict of interest.

## Footnotes

1 http://rsb.info.nih.gov/ij/

2 www.R-project.org

## Abbreviations

1MOI3M: 1-methoxyindol-3-ylmethyl glucosinolate
4MOI3M: 4-methoxyindol-3-ylmethyl glucosinolate
4-MSOB: 4-methylsulfinylbutyl glucosinolate
4-MTB: 4-methylthiobutyl glucosinolate
5-MSOP: 5-methylsulfinylpentyl glucosinolate
6-MSOH: 6-methylsulfinylhexyl glucosinolate
7-MSOH: 7-methylsulfinylheptyl glucosinolate
7-MTH: 7-methylthioheptyl glucosinolate
8-MSOO: 8-methylsulfinyloctyl glucosinolate
8-MTO: 8-methylthiooctyl glucosinolate
APK: APS kinase
APR: APS reductase
APS: adenosine 5′-phosphosulfate
ATPS: ATP sulfurylase
BAW: beet armyworm
DMDS: dimethyl disulfide
BCAT: branched-chain amino acid amino transferase
CFU: colony forming unit
CYP: cytochromes P6_450_
DDW: double-distilled water
ESI-MS: electrospray ionization-mass spectrometry
FMO_GS-OX_: flavin monooxygenase
GST: glutathione-*s*-transferase
I3M: indol-3-ylmethyl glucosinolate
IGMT: indole glucosinolate methyl transferase
IPMDH: isopropyl malate dehydrogenase
IPMI: isopropyl malate isomerase
MAM: methylthioalkyl malate synthase
MSG: methylsulfinylalkyl glucosinolates
MTG: methylthioalkyl glucosinolates
PAPS: 3′-phosphoadenosine 5′-phosphosulfate
PDA: photodiode array
PGPR: plant growth-promoting rhizobacteria
RT-PCR: reverse-transcription PCR
SOT: sulfotransferase
SUR: super root
TSA: tryptic soy agar
UHPLC: ultra-HPLC
VOCs: volatile organic compounds
