# Supplementary material for: Augmenting Sulfur Metabolism and Herbivore Defense in Arabidopsis by Bacterial Volatile Signaling
Source: Front Plant Sci. 2016 Apr 8;7:458. doi: 10.3389/fpls.2016.00458 (PMC4824779; doi:10.3389/fpls.2016.00458)
Supplement: Supplementary file 1 [file Table_1.DOCX]

**Supplemental Data**


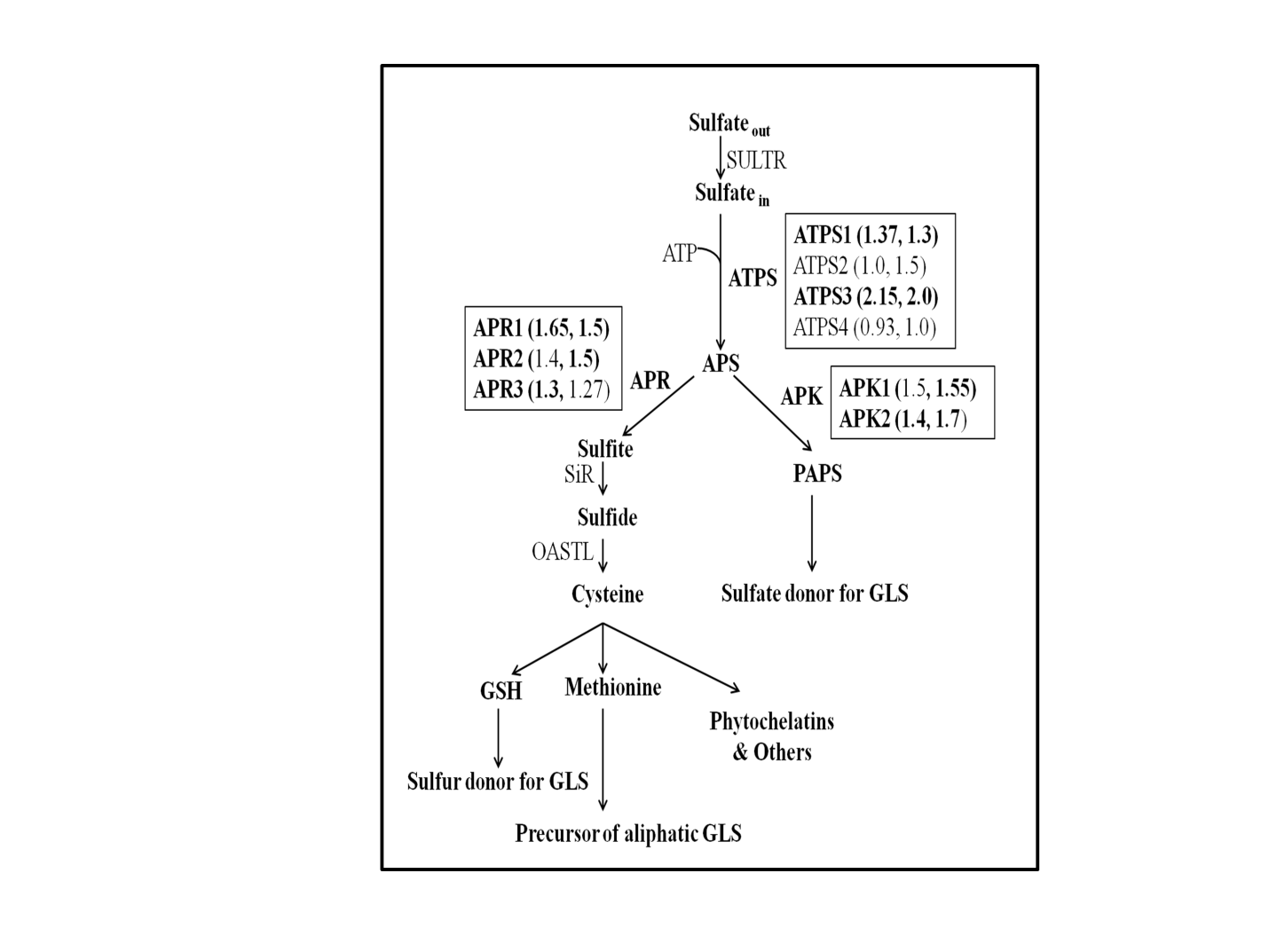


**Suppl. Fig. 1.** Induced sulfate assimilation genes with fold change in brackets at 48 and 72 hours post GB03 exposure based on whole-plant microarray data (Zhang et al., 2007); bold values indicate statistically-significant difference between treatments (*t*-test, *P* value ≤ 0.05, *n* = 3).

.


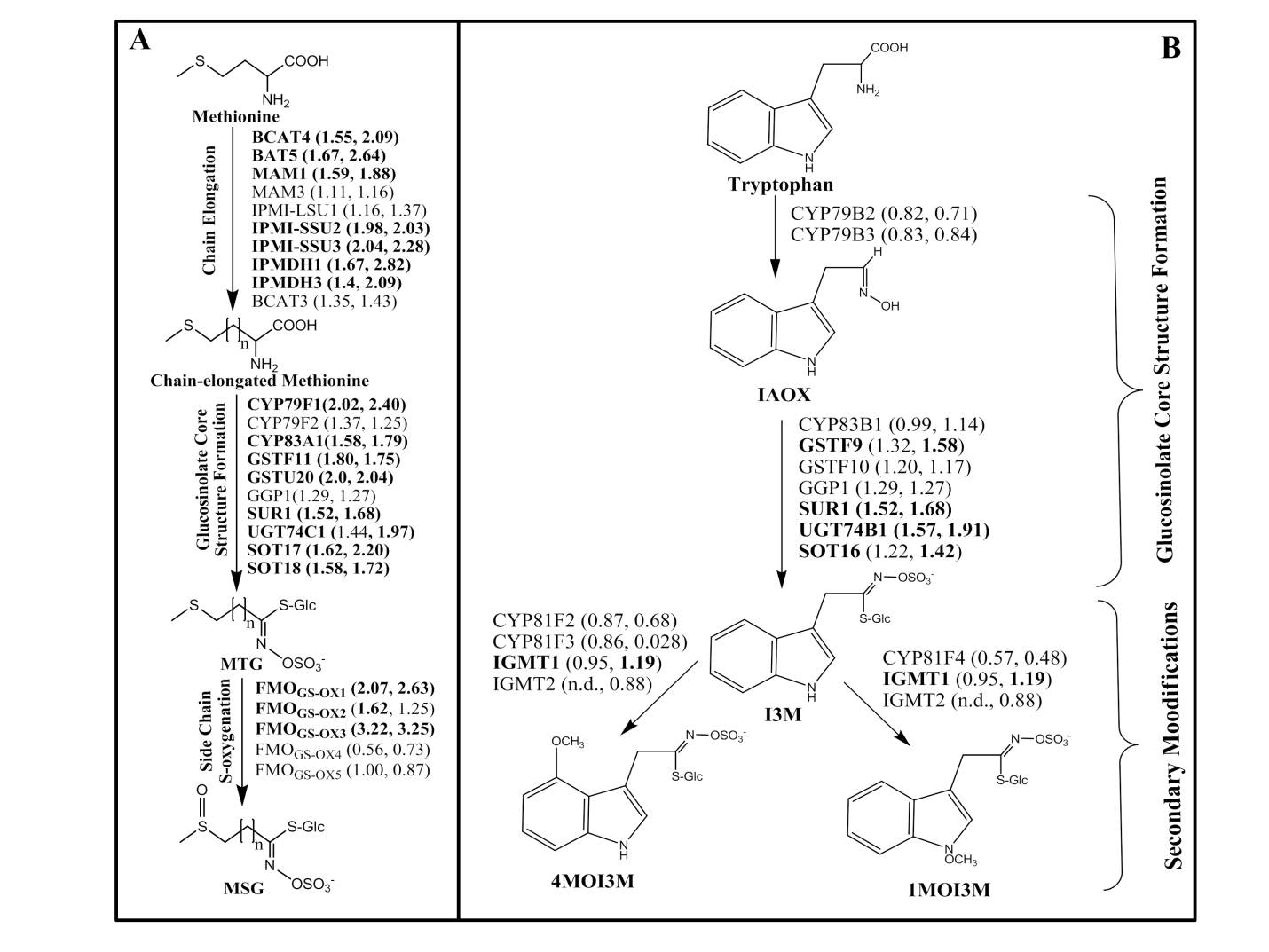


**Suppl. Fig. 2.** Aliphatic **[A]** and indolic **[B]** glucosinolate biosynthetic genes with fold change in brackets at 48 and 72 hours post GB03 exposure based on whole-plant microarray data (Zhang et al., 2007); bold values indicate statistically-significant difference between treatments (*t*-test, *P* value ≤ 0.05, *n* = 3).


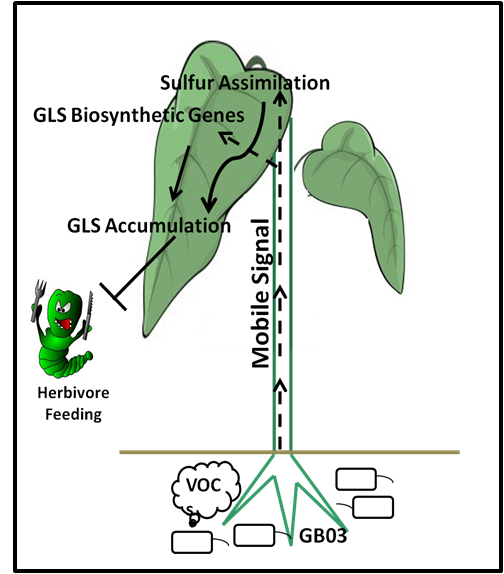


**Suppl. Fig. 3.** Proposed model for GB03-conferred protection against herbivore feeding. GB03 root colonization, via direct interaction and/or volatile emission, triggers a yet unidentified signaling pathway resulting in transcriptional up-regulation of sulfur assimilation and glucosinolate biosynthetic genes that leads to increase in glucosinolates accumulation and plant protection against herbivore damage. Dashed arrows are proposed steps not confirmed in the current study.
